# Nephroprotective role of resveratrol in renal ischemia-reperfusion injury: a preclinical study in Sprague-Dawley rats

**DOI:** 10.1186/s40360-024-00809-8

**Published:** 2024-10-28

**Authors:** Elaf R. Alaasam, Ali M. Janabi, Karrar M. Al-Buthabhak, Rihab H. Almudhafar, Najah R. Hadi, Athanasios Alexiou, Marios Papadakis, Mohammed E. Abo-El Fetoh, Dalia Fouad, Gaber El-Saber Batiha

**Affiliations:** 1 Alsadar Medical City, Directorate of Najaf Health, Najaf, Iraq; 2 Department of Pharmacology and Toxicology, Faculty of Pharmacy, University of Kufa, Najaf, Iraq; 3 Department of Internal Medicine, Faculty of Medicine, University of Kufa, Najaf, Iraq; 4 Department of Pathology and Forensic Medicine, Faculty of Medicine, University of Kufa, Najaf, Iraq; 5 Department of Pharmacology and Therapeutics, Faculty of Pharmacy, University of Kufa, Najaf, Iraq; 6 Department of Research & Development, Funogen, Athens, 11741 Greece Attiki; 7 University Centre for Research & Development, Chandigarh University, Chandigarh-Ludhiana Highway, Mohali, Punjab, India; 8 Department of Surgery II, University Hospital Witten-Herdecke, University of Witten-Herdecke, Heusnerstrasse 40, 42283 Wuppertal, Germany; 9 Department of Pharmacology and Toxicology, Faculty of Pharmacy, Egyptian Russian University, Cairo, Egypt; 10 Department of Zoology, College of Science, King Saud University, PO Box 22452, Riyadh, 11495 Saudi Arabia; 11 Department of Pharmacology and Therapeutics, Faculty of Veterinary Medicine, Damanhour University, Damanhour, AlBeheira 22511 Egypt

**Keywords:** Renal Ischemia-Reperfusion Injury, Nephroprotection, Resveratrol, Oxidative Stress, Inflammation, Apoptosis

## Abstract

**Background:**

Renal ischemia-reperfusion injury (IRI) is a significant contributor to renal dysfunction, acute kidney injury (AKI), and associated morbidity and mortality. Resveratrol, a polyphenol and phytoalexin, is known for its anti-inflammatory, antioxidant, and anti-cancer properties. This study investigates the nephroprotective potential of resveratrol in a rat model of renal IRI.

**Materials and methods:**

Twenty-eight male Sprague-Dawley rats were divided into four groups: *Sham*, *IRI*, *DMSO*, and *Resveratrol*. The *Sham group* underwent identical procedures without renal pedicle clamping, while the *IRI group* experienced 30 min of ischemia followed by 2 h of reperfusion. The *DMSO group* received dimethyl sulfoxide (DMSO) intraperitoneally 30 min before ischemia, and the *Resveratrol group* received 30 mg/kg resveratrol intraperitoneally 30 min before ischemia. Biochemical parameters (Urea, creatinine, IL-1β, NF-κβ, SOD, GSH, Bcl-2, and caspase-3) and histopathological changes were assessed.

**Results:**

IRI caused a substantial increase in serum creatinine, Urea, IL-1β, NF-κβ, and caspase-3 levels, while simultaneously decreasing SOD, GSH, and Bcl-2 levels. Resveratrol treatment mitigated these effects by lowering inflammatory and apoptotic markers, enhancing antioxidant defenses, and improving histological outcomes.

**Conclusion:**

Resveratrol demonstrates significant nephroprotective effects in renal IRI, primarily through its antioxidant, anti-inflammatory, and anti-apoptotic properties.

## Introduction

Ischemia/reperfusion injury (IRI) occurs when an organ’s blood supply is cut off briefly before being restored along with its oxygen supply. The activation of leukocytes, infarction, sepsis, and the generation of reactive oxygen species (ROS) all contribute to the worsening of tissue damage after a transplant, infection, or other medical procedure [1].

Differentiating between the two phases of the syndrome in IRI is crucial because they are separate but related. The terms “ischemia” and “reperfusion” describe the period during which blood flow is restricted or interrupted and when blood flow is restored and oxygen enters the organ. Ischemic and reperfusion insults are distinct and elicit different organ/cell responses, and together, they add up to total IRI damage [2]. I/R may cause a variety of symptoms in the body, including a weakened heart, reduced brain function, reperfusion arrhythmias, a gastrointestinal barrier collapse, and, the most dangerous of all, multiorgan dysfunction syndrome [3].

The combined occurrence of renal ischemia and perfusion damage (RIRI) during a kidney transplant increases the risk of complications, death, and hospitalization duration. RIRI is a leading cause of kidney failure and acute kidney damage [4]. Certain circumstances, such as hypotension and shock, may cause RIRI. Renal failure may occur in rare cases due to thrombosis or dissection of the primary renal artery [5]. Acute kidney injury (AKI) occurs in the kidneys when blood flow is restricted (renal ischemia), leading to a shortage of oxygen (renal hypoxia). The incidence of morbidity and mortality rises as the glomerular filtration rate and renal output fall in tandem [6]. The development process of renal ischemia/reperfusion (I/R) is intricate, including many pathogenic pathways, including neutrophil activation, generation of reactive oxygen species (ROS), adhesion molecules, and a diverse array of cytokines [7, 8]. Multiple RIRI hallmarks promote renal tubular epithelial and endothelial cell dysfunction and tissue-resident leukocyte activation. Without ATP, glycogen, or oxygen, DNA damage, vascular leakage, immunological activation, endothelial cell activation, and leukocyte adherence are hallmarks. Consequently, there is an increase in vascular leakage and interstitial edema [9, 10].

Resveratrol is a polyphenol and phytoalexin found in many fruits and vegetables, including red wine, plums, olive oil, peanuts, cranberries, berries, and grapes [11]. It has been found in various fungi, including *Botryosphaeria* and *Penicillium*. *Polygonum cuspidatum*, a traditional Chinese medicine plant, was the first to have resveratrol isolated from it [12]. Resveratrol is found to have numerous biological activities in both the cis and trans configurations. Though trans-resveratrol has been given more credit for its potential than its cis isomer, it is converted to cis-resveratrol upon exposure to ultraviolet light [13, 14]. Resveratrol is a powerful antioxidant with anti-inflammatory, anti-aging, cardiovascular, protective, diabetic, and cancer-fighting properties [15].

This study investigates the nephroprotective potential of resveratrol in a rat model of renal IRI. Moreover, it introduces a novel methodology by employing resveratrol alongside (Urea, creatinine, IL-1β, NF-κβ, SOD, GSH, Bcl-2, and caspase-3), providing a new perspective on AKI. This combination has not been previously explored, making these findings particularly significant in advancing RIRI to complete the current literature evidence.

## Methods

### Animals’ preparation

From the Faculty of Science at the University of Kufa, we obtained 28 male Sprague Dawley rats ranging in weight from 150 to 250 g and aged 15 to 20 weeks. The rats lived in the animal facility of the Faculty of Pharmacy at the University of Kufa. The animals were housed in a separate room on a 12-h light/12-h dark cycle, with a temperature and humidity of 24 ± 2 degrees Celsius and 60–65%, respectively. The rats received a standard diet of food and water. All experimental protocols were approved by the Institutional Animal Care and Use Committee (IACUC) at Kufa University after submitting the required applications (**khq/6019**,** 27/02/2023**).

### Study design

Randomization was used to divide 28 adult male Sprague-Dawley rats into four groups (*n* = 7 in each): **Sham**, **IRI**, **DMSO**, and **Resveratrol**. The kidneys in the **sham group** underwent the same procedures as those in the **IRI group** without clamps on the renal pedicles. In contrast, those in the **IRI group** underwent a midline laparotomy and had 30 min of ischemia followed by 2 h of reperfusion. The **DMSO-group rats** received DMSO vehicles for resveratrol by intraperitoneal injection 30 min before ischemia [16]. In the **resveratrol group**, 30 mg/kg of resveratrol was injected intraperitoneally 30 min before the onset of I/R [17, 18]. For the duration of the operation, the rats were given an intraperitoneal injection of 100 mg/kg ketamine and 10 mg/kg xylazine [19, 20].

### Preparation of the drug

Resveratrol has a solubility of 100 mg/mL in DMSO, the standard vehicle, according to the instruction leaflet of the manufacturing company, and should be prepared immediately before use (MedChem Express company instructions). The dose was administered intraperitoneally, according to body weight [17].

### Sampling techniques

#### Blood sample collection

Initially, the animals were decapitated followed by removing about 2–4 ml of blood from the heart [19, 20]. The blood sample was collected without anticoagulant in a gel tube and centrifuged at 6000 rpm for 10 min to separate the serum for the measurement level of Urea, creatinine, NF-κβ, and IL-1β using commercial ELISA kits.

#### Tissue sampling for biochemical analysis

The kidney tissues were stored at −80 °C until homogenization with a high-intensity ultrasonic liquid processor in 1:10 W/V phosphate buffered saline containing 1% Triton X-100 and a protease inhibitor cocktail [21]. Homogenate was centrifuged for 10 min at 5000 rpm at 4 °C, and using the supernatants, we were able to test for GSH, SOD, caspase-3, and Bcl-2 using available ELISA kits.

#### Tissue sampling for histopathology

The kidney tissue section was fixed in 10% formaldehyde, dehydrated in alcohol series, cleared in xylene, and embedded in paraffin. Kidney tissues were paraffin-embedded and then sectioned into 5-m-thick slices. Haematoxylin and eosin were then used to stain the sections [22]. Damaged cells were examined in 5 non-overlapping views, and a score was given.

### Investigations

#### **Biochemical analysis of renal function**

Urea and creatinine concentrations were determined using commercially available Elisa kits (urea Elisa kit from SunLong Biotech Co., Ltd., China, Cat. No. SL1053Ra) and creatinine Elisa kit from Bioassay Technology Laboratory, China, Cat. No. EA0116Ra) and their respective manufacturer guidelines.

#### Analysis of NF-κβ, and IL-1β

The levels of NF-κβ and IL-1β were measured using NF-κβ and IL-1β Elisa kits obtained from Bioassay Technology Laboratory, China, Cat. No. E0290Ra, Cat. No. E0119Ra respectively as stated in the manufacturer’s directions.

#### Analysis of GSH and SOD

The levels of GSH and SOD were measured using GSH and SOD Elisa kits obtained from Bioassay Technology Laboratory, China, Cat. No. EA0113Ra, Cat. No. E0168Ra, respectively, along with the manufacturer’s instructions.

#### Analysis of Caspase-3 and Bcl-2

The levels of Caspase-3 and Bcl-2 were measured using Caspase-3 and Bcl-2 Elisa kits obtained from Bioassay Technology Laboratory, China, Cat. No. E1648Ra, Cat. No. E0037Ra, respectively, consistent with the manufacturer’s information.

### Statistical analysis

Analyses were done using SPSS (Statistics Package for the Social Sciences) Form 27. Data were displayed as mean ± standard error mean. The analysis of variance, one-way ANOVA, was employed, followed by post-hoc analysis using the LSD method. Changes in histopathology were compared between groups using a combination of Fisher’s exact test and the Kruskal-Wallis one-way analysis of variance. The statistically significant level was set at *P* < 0.05 in all tests.

## Results

Ischemia was maintained for 30 min, and then reperfusion was induced for 2 h. Thirty minutes before ischemia, the animals were pre-treated with **DMSO** (as a vehicle), **Resveratrol** (30 mg/kg), or left untreated (**Sham** and **IRI** groups). Various biochemical and histopathological parameters were investigated to evaluate the extent of the injury.

### Effect on renal function parameters

**Fig. 1:**
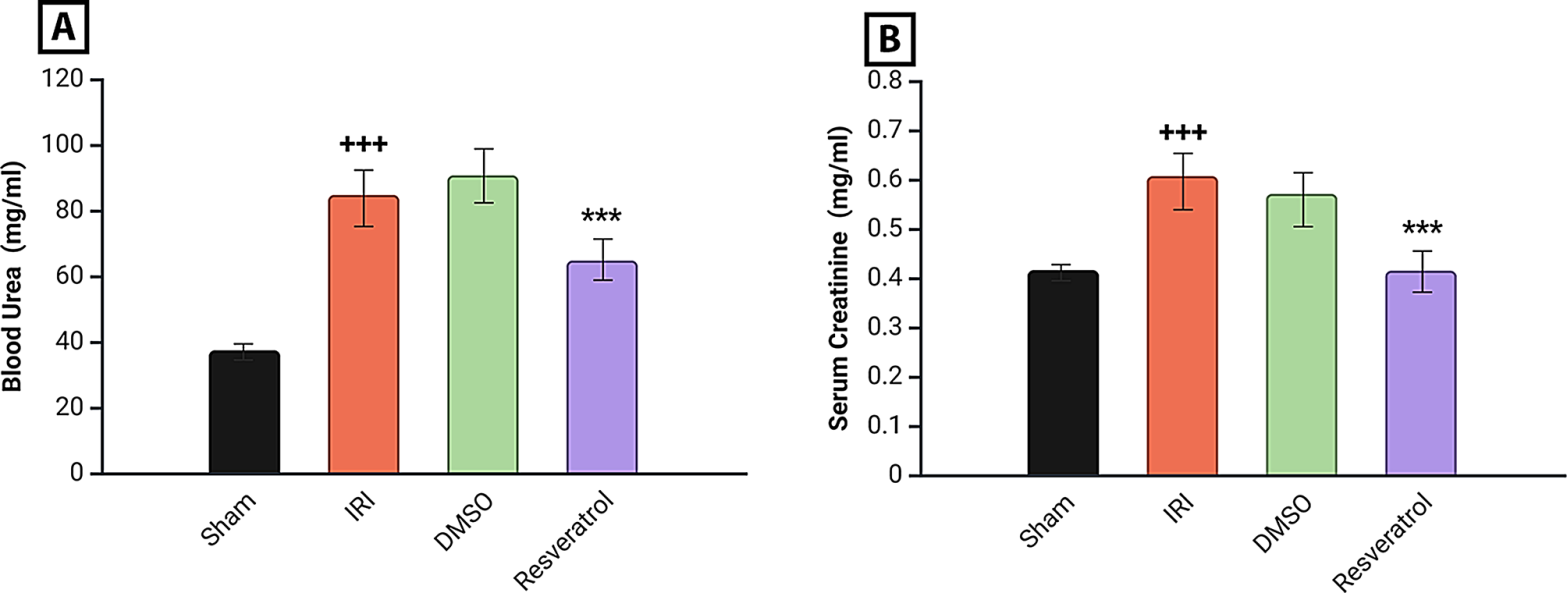
Renal function analysis. **A** Serum urea level of study groups, **B** Serum creatinine level of study groups. Rats were subjected to ischemia for 30 min and reperfusion for 2 h. Rats were pre-treated with either vehicle DMSO, resveratrol (30 mg\kg), or left untreated (sham and induced group) 30 min before ischemia. Urea and Cr concentrations were determined using Urea and Cr ELISA kits. One-way ANOVA followed by an LSD multiple comparison test was used for analysis. Data are presented as mean ± SEM. ^**+++**^*P* < 0.001 vs. sham group, ***P < 0.001, **P < 0.01 vs. induced group

After renal I/R, marked elevations in serum Cr and Urea were noticed in the **IRI group** compared to the **Sham group**. Serum Cr and Urea levels dropped dramatically after resveratrol treatment (Fig. 1).

### Effect on serum inflammatory parameters

**Fig. 2:**
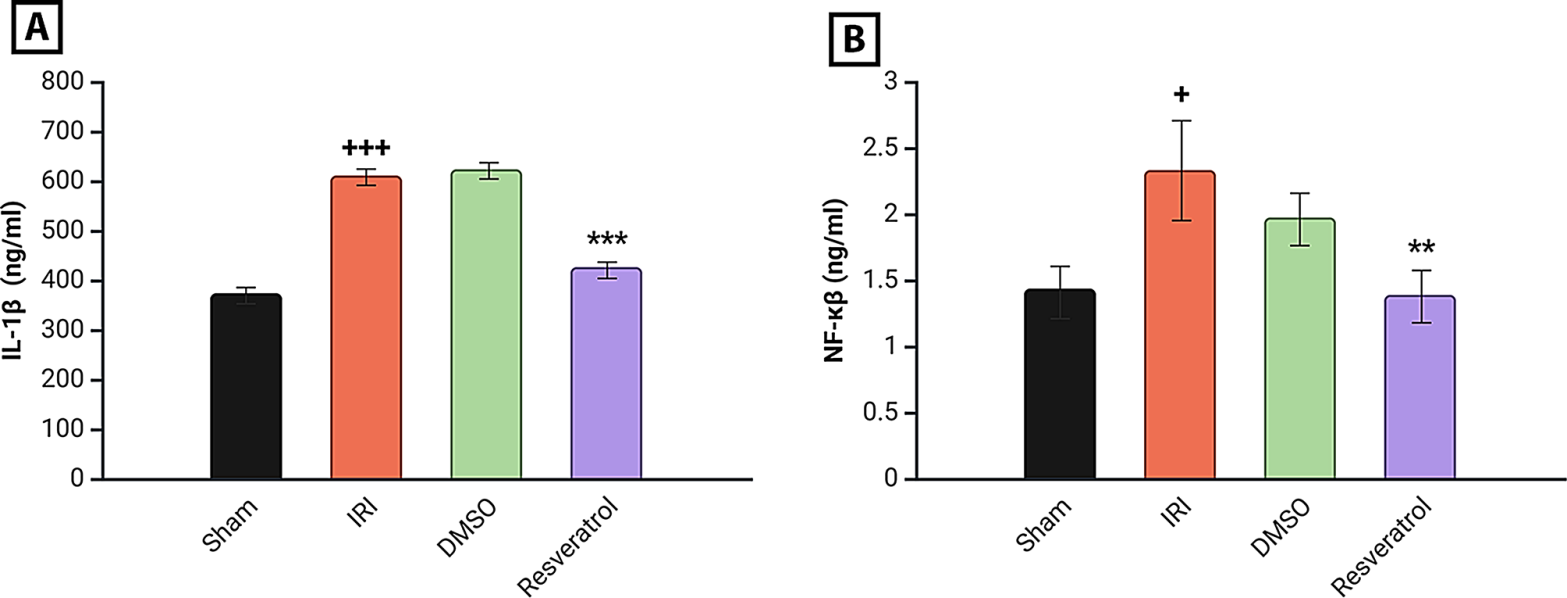
Renal inflammatory markers analysis. **A** IL-1B level of study groups, **B** NF-κβ level of study groups. Rats were subjected to ischemia for 30 min and reperfusion for 2 h. Rats were pre-treated with either vehicle DMSO, resveratrol (30 mg\kg), or left untreated (sham and induced group) 30 min before ischemia. The concentration of IL-1B and NF-κβ levels was determined using ELISA kits. The data was analyzed using one-way ANOVA and the LSD multiple comparison test for further interpretation. Data are shown as mean ± SEM, ^**+++**^*P* < 0.001 vs. sham group, ***P < 0.001 vs. induced group, ^+^*P* < 0.05 vs. sham group, **P < 0.01 vs. induced group

Marked elevations in serum IL-1β and NF-κβ were observed in the induced group when compared to the sham group. The resveratrol group significantly lowered serum inflammatory markers (IL-1β, NF-κβ) (Fig. 2).

### Effect on oxidative stress and apoptotic markers

**Fig. 3:**
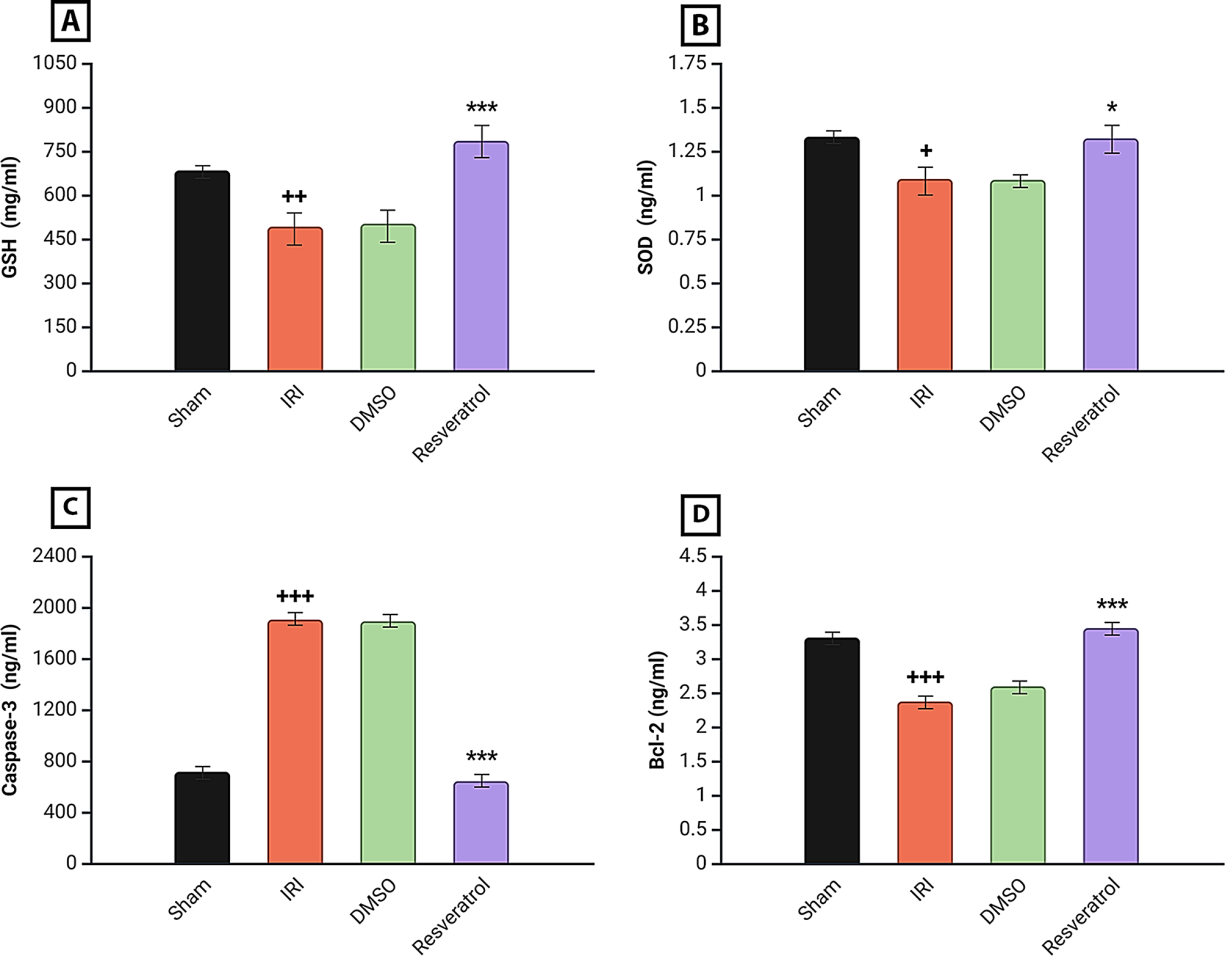
Renal oxidative stress and apoptotic markers analysis. **A** GSH level, **B** SOD level, **C** Caspase-3 level, **D** Bcl-2 level. Rats were subjected to ischemia for 30 min and reperfusion for 2 h. Rats were pre-treated with either vehicle DMSO, resveratrol (30 mg\kg), or left untreated (Sham and induced group) 30 min before ischemia. The concentration of GSH, SOD, Caspase-3, and Bcl-2 levels was determined using ELISA kits. LSD multiple comparison test was used after One-way ANOVA for analysis. Information is displayed as mean ± SEM, ^+^*P* < 0.05 vs. sham group, ^**++**^*P* < 0.01 vs. sham group, *P < 0.05 vs. induced group, ***P < 0.001 vs. induced group, ^**+++**^*P* < 0.001 vs. sham group

Marked elevations in Caspase-3 with SOD, GSH, and Bcl-2 decreases were substantially noticed in the renal tissue of rats in the **IRI group** when compared to the **Sham group**. With **resveratrol** treatment, renal tissue levels of antioxidant markers (SOD, GSH) were significantly increased, apoptotic marker (Caspase-3) levels were markedly decreased, and anti-apoptotic marker (Bcl-2) levels were significantly increased in comparison to the **IRI group** (Fig. 3).

### Effect on renal damage score

**Fig. 4:**
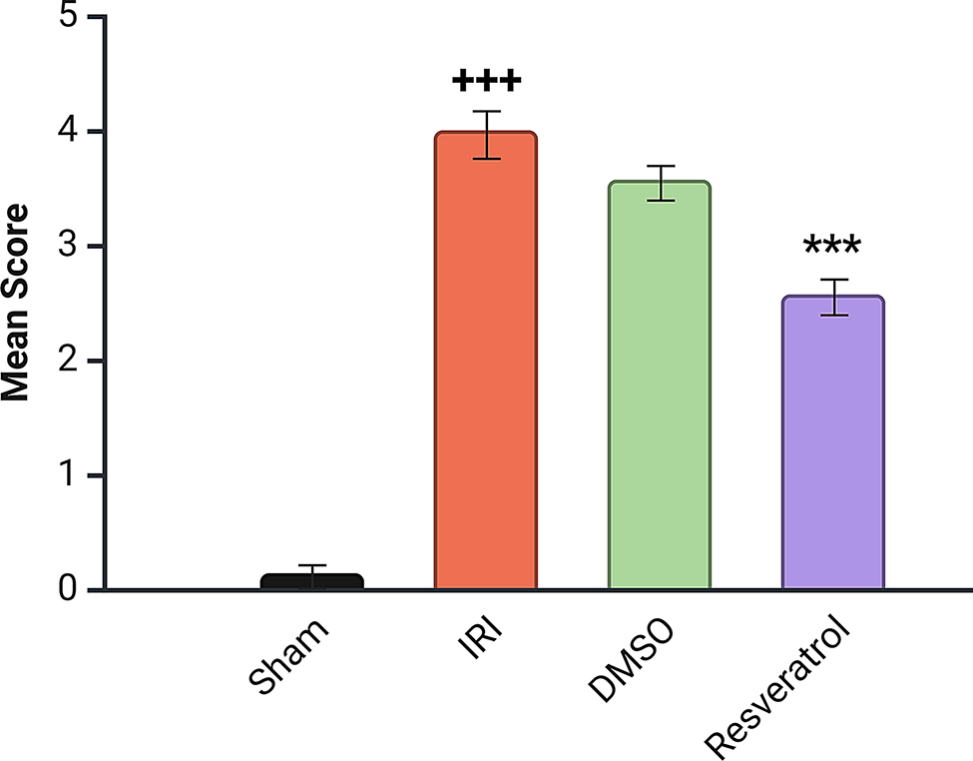
Mean histopathological renal tubular damage score ^+++^*p* < 0.001 vs. sham group, ***p < 0.001 vs. induced group. Kruskal-Wallis test was used for analysis; data are presented as mean ± SEM. ^**+++**^*P* < 0.001 vs. sham group, ***P < 0.001 vs. induced group

The **Sham group** has significantly lower damage scores compared to the other groups (+++ *p* < 0.001 vs. Sham). The **resveratrol group** shows considerably less damage than the **IRI** and **DMSO** groups (***p <0.001 vs. induced groups), indicating a nephroprotective effect (Fig. 4). Furthermore, Table 1 shows how the renal damage varies between the groups and highlights the nephroprotective effect of resveratrol. The **Sham group** shows minimal damage across all parameters, with a total lesion score close to 1, representing normal renal structure. Severe renal damage is observed, with scores close to 4 for all parameters, resulting in a total score of 11.8, representing extensive tissue damage in the **IRI group**. Similar to the **IRI group**, **DMSO** alone does not protect against renal injury. Interestingly, the **resveratrol group** showed moderate protection, with scores around 2 for each parameter and a total lesion score of 5.9, reflecting partial renal recovery.

**Table 1:** Lesion-Scoring table

Group	Tubular necrosis	Hyalinization	Apoptosis	Total lesion score
Sham	0.5	0.2	0.3	1.0
IRI	4.0	3.8	4.0	11.8
DMSO	3.9	3.7	3.8	11.4
Resveratrol	2.0	1.8	2.1	5.9

### Effect on histopathological examination

**Fig. 5:**
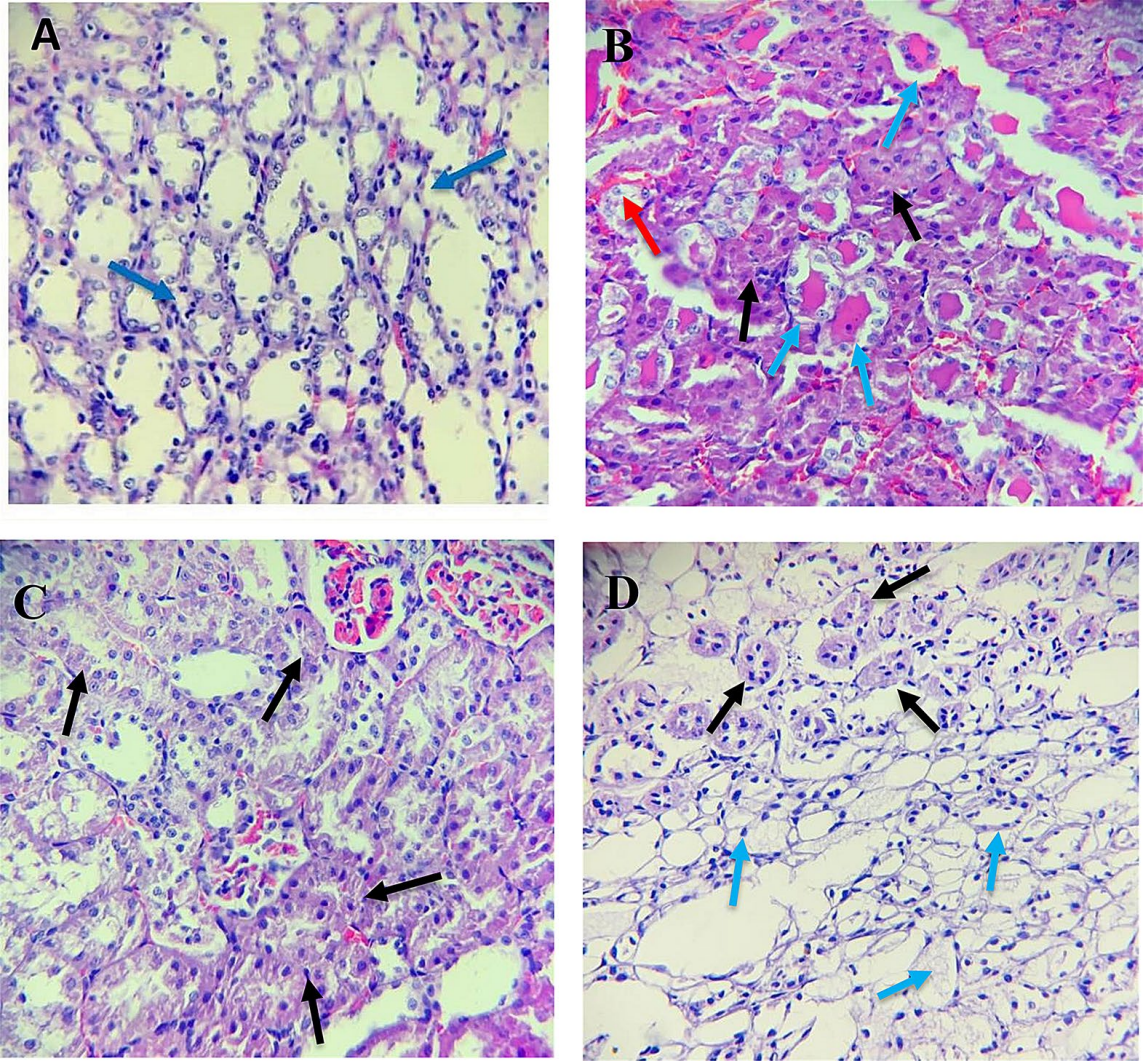
Histopathological examination of kidney tissues. **A *Sham group***, normal histology of renal tubules (*blue arrows*). **B *IRI group***, renal tubules with score 4 damage. Cellular swelling increased cytoplasmic eosinophilia (*black arrows*), eosinophilic cast (*blue arrows*) & hemorrhage (*red arrow*). **C *DMSO group***, renal tubules with score 4 damage. Cellular swelling increased cytoplasmic eosinophilia (*black arrows*). **D *Resveratrol group***, renal tubules with score 2 damage. Cellular swelling increased cytoplasmic eosinophilia, involving 40% of the examined tubules (*black arrows*) and normal tubules (*blue arrows*). (H&E x400)

The **Sham group** had a typical histology of renal tubules. The **IRI group** had renal tubules with severe damage, characterized by cellular swelling, heightened cytoplasmic eosinophilia, the presence of eosinophilic casts, and bleeding. The **DMSO group** had a pathology that was comparable to the **IRI group**. In the group that received **resveratrol**, renal tubules with a damage score 2 exhibited cellular swelling, increased cytoplasmic eosinophilia affecting 40% of the observed tubules, and regions of unaffected tubules (Fig. 5).

## Discussion

Acute kidney injury (AKI) is a common illness, particularly among patients who are hospitalized. It may occur in around 7% of hospital admissions and up to 30% of intensive care unit admissions [10]. This research aims to clarify the protective effects of resveratrol on the kidneys, specifically its potential to maintain renal function, alleviate inflammation, decrease oxidative stress, and prevent cell death in the setting of ischemia-reperfusion damage (IRI). As shown in the current study, renal ischemia/reperfusion increased serum Cr, BUN, IL-1β, NF-κβ, and Caspase-3 in rats, while SOD, GSH, and Bcl-2 were reduced. Interestingly, treatment with resveratrol reduced Cr and BUN levels, reduced inflammatory markers (IL-1β, NF-κβ), apoptotic marker (Caspase-3), increased antioxidant (SOD, GSH) and anti-apoptotic markers (Bcl-2), and improved renal histological scores. The latest findings demonstrate that resveratrol reduces the increase in blood creatinine and urea levels, in line with prior research conducted by Xiao et al. [23], which showed that resveratrol administration considerably improved kidney function.

An essential component of renal ischemia-reperfusion injury (IRI) is the inflammatory reaction, which worsens tissue harm and adds to renal impairment. Therapeutically inhibiting this inflammatory cascade is a viable approach to safeguard renal tissue. RIRI stimulates the production of pro-inflammatory cytokines, such as IL-1β, TNF-α, and IL-6, while decreasing anti-inflammatory cytokines like IL-10. These pro-inflammatory cytokines enhance the local inflammatory response and sustain tissue damage by attracting more inflammatory mediators [24].

Chronic renal failure patients and animal models of acute kidney injury (AKI) caused by nephrotoxic drugs have shown increased levels of IL-6, IL-1β, and TNF-α [25]. Recent studies have emphasized the crucial importance of the NF-κβ signaling pathway in causing inflammatory damage during renal ischemia. More precisely, the activation of NF-κβ, triggered by the breakdown of its inhibitor IκB, results in the transcription of genes that encode pro-inflammatory cytokines [26, 27]. Our research discovered that administering resveratrol before treatment significantly decreased the levels of IL-1β and NF-κβ in kidney tissues, which is consistent with the results reported by Wang et al. [28]. In their study, resveratrol substantially inhibited TNF-α-induced NF-κβ signaling in diabetic rats undergoing renal ischemia/reperfusion [28].

Oxidative stress is a significant factor in the development of renal ischemia/reperfusion (I/R) injury. It occurs due to an excessive generation of reactive oxygen species (ROS), which leads to lipid peroxidation, protein oxidation, and DNA damage. The resulting oxidative stress triggers apoptosis and cellular death, impairing the functionality of the kidneys. The disparity between the creation of reactive oxygen species (ROS) and the body’s ability to defend against them with antioxidants is crucial in renal ischemia-reperfusion injury (RIRI). This injury is worsened by the reduction in activity of necessary antioxidant enzymes such as catalase, superoxide dismutase (SOD), and glutathione peroxidase, leading to increased tissue damage [29]. The current research found that resveratrol pre-treatment considerably increased the levels of superoxide dismutase (SOD) and glutathione (GSH) in renal tissues, highlighting its potent antioxidant effects.

These results align with previous research that has emphasized resveratrol’s capacity to diminish oxidative stress and inhibit the decline of antioxidant enzymes in models of renal ischemia/reperfusion damage [28, 30, 31]. In addition, the literature extensively documents the mechanisms by which resveratrol provides renoprotection, including its ability to scavenge radicals and prevent neutrophil infiltration [31]. Research on resveratrol analogs, including RSVA405 and RSVA314, has shown that they may effectively regulate energy metabolism, oxidative stress, and inflammation [32]. These findings emphasize the compound’s diverse protective properties [33].

The use of nanoparticles to transport resveratrol has been shown to prevent cell death and reduce inflammation effectively, reinforcing its promise as a treatment for renal ischemia-reperfusion injury [34]. In addition, Li et al. [22] conducted western blot studies, which validated that resveratrol’s renoprotective benefits are achieved by inhibiting oxidative stress, reducing apoptosis, and suppressing inflammatory responses.

Apoptosis is a crucial process in renal ischemia-reperfusion injury (IRI), where the activation of caspase-3 is an essential step for carrying out programmed cell death. The equilibrium between pro-apoptotic (Bax) and anti-apoptotic (Bcl-2) proteins is vital for the survival of cells. Ischemia-reperfusion injury (IRI) often disrupts this equilibrium by upregulating Bax expression and downregulating Bcl-2 levels, promoting apoptosis [35]. This work proved that pre-treatment with resveratrol effectively reduced caspase-3 activity and restored Bcl-2 levels in damaged renal tissues. These findings support the conclusion that resveratrol can prevent apoptosis after renal ischemia/reperfusion injury. The findings align with those of Wang et al. [28], who reported comparable beneficial effects in diabetic rats who were administered resveratrol as a pre-treatment.

Additional histopathological investigations provided further evidence of the protective effects of resveratrol on the kidneys. These analyses showed a substantial reduction in tissue damage, including lower infiltration of inflammatory cells, dilatation of renal tubules, and edema in rats treated with resveratrol compared to the group of rats not treated with resveratrol but subjected to ischemia-reperfusion injury (IRI). The observations above support prior research that demonstrates the protective effects of resveratrol on the structure of the kidneys and the reduction of cellular harm in models of renal ischemia/reperfusion injury [22, 30].

Aside from resveratrol, moxipril [36] and erythropoietin [37] have also exhibited comparable protective effects against RIRI, thus confirming the efficacy of antioxidant and anti-inflammatory approaches in reducing renal I/R injury.

## Conclusion

Resveratrol has been shown to have a nephroprotective effect against renal damage caused by ischemia/reperfusion (I/R). Resveratrol exhibited antioxidant, anti-inflammatory, and anti-apoptotic characteristics by controlling oxidative stress, suppressing inflammation, and reducing apoptotic markers after renal ischemia/reperfusion damage.

## Data Availability

No datasets were generated or analysed during the current study.
